# Flu vaccination among older persons: study of knowledge and practices

**DOI:** 10.1186/s41043-018-0159-8

**Published:** 2019-01-03

**Authors:** Tatjana Gazibara, Nikolina Kovacevic, Darija Kisic-Tepavcevic, Selmina Nurkovic, Ilma Kurtagic, Teodora Gazibara, Tatjana Pekmezovic

**Affiliations:** Institute of Epidemiology, Faculty of Medicine, University of Belgrade, Visegradska 26A, Belgrade, 11000 Serbia

**Keywords:** Flu, Vaccine, Older persons, Knowledge

## Abstract

**Background:**

Surveys of older adults’ knowledge relative to flu immunization have highlighted its relevance in the improvement of vaccination coverage. The purpose of this study was to estimate the proportion of older persons who have ever been immunized against seasonal flu in the municipality of Vračar (Belgrade, Serbia), assess their knowledge related to flu and flu vaccination, and to determine factors associated with flu immunization.

**Methods:**

In the period November 2012–January 2013, 480 persons aged above 65 years were recruited at the Community Health Center, of which 354 persons were finally included in the study. Data were collected using a questionnaire. To assess the level of knowledge, correct answers were awarded 1 point. The total knowledge score ranged from 0 to 17 and was divided into three levels: poor (0–4 points), moderate (5–8 points), good (9–13 points), and excellent (14–17 points).

**Results:**

The proportion of ever vaccinated older persons was 47.7%. Of those, one third (29.1%) had been immunized regularly. Most seniors (61.9%) demonstrated good, whereas one third (29.8%) demonstrated excellent level of knowledge. In terms of reasons for non-compliance, the highest proportion of older persons declined vaccination because “they were in good health” (33.5%) and because “they did not believe that vaccine protects from flu” (31.5%). Independent predictors of being ever immunized against seasonal flu were having higher level of education, being more knowledgeable relative to flu vaccination, and taking more medications.

**Conclusions:**

Our results indicate that health care sector requires well-coordinated promotion campaigns to enhance acceptance of flu vaccination. Organized immunization counseling could provide accurate, evidence-based information in order to transform misbeliefs, prejudice, and negative attitude towards vaccination.

**Electronic supplementary material:**

The online version of this article (10.1186/s41043-018-0159-8) contains supplementary material, which is available to authorized users.

## Background

Flu is an acute respiratory infection, caused by three types of flu viruses, influenza virus A, B, and C. Type A affects humans as well as animals (birds, pigs) and causes moderate to severe illness in all age groups [1, 2]. Flu symptoms occasionally worsen, leading to the onset of pneumonia [3]. Flu epidemics cause three to five million cases of severe illness, while up to 500,000 persons die as a result [2]. The majority of flu-related deaths in industrialized countries occur in persons over 65 years of age [2]. Additionally, the increase in flu mortality rates has been associated with complications of the existing chronic conditions [4–6]. Because of the increase in health care costs, flu represents a major public health issue [2]. Therefore, vaccination against flu is the most effective preventive measure, although it has been noted that vaccine effectiveness may vary across seasons as well as across different age and population groups [7].

Flu outbreaks exhibit typical seasonal patterns, both in Northern and Southern Hemispheres [8]. In the Republic of Serbia, seasonal flu vaccination begins at the end of October, while the costs of immunization are covered by health insurance. Immunization is organized and carried out in Community Health Centers, when trivalent inactivated flu vaccine is administered intramuscularly. According to law, vaccination is compulsory for persons suffering from chronic pulmonary and cardiovascular diseases, diabetes, and other metabolic disorders as well as for persons residing in assisted living facilities, health-care workers, and persons over the age of 65 [9]. Therefore, around 350,000 individuals require flu immunization in the capital city, Belgrade [9]. However, despite regulations, those who refuse flu vaccination do not bear any legal consequences. After completion of vaccination for the season 2011/2012, it has been observed that persons over 65 years of age account for the highest proportion of immunized individuals [10].

Surveys of older adults’ knowledge relative to flu immunization have highlighted its relevance in the improvement of vaccination coverage [11, 12]. Yap et al. [12] have indicated that knowledge has a significant influence on attitudes and practices of flu immunization. Still, socio-cultural dimensions should be taken into consideration when evaluating flu vaccine acceptance. Specifically, a systematic review of determinants of flu vaccine uptake reported that individual’s misconceptions attributable to poor knowledge and personal beliefs have negative impact on flu vaccination compliance in older adult population [13]. Moreover, even though past flu vaccinations tend to be predictive of future vaccination [14], vaccine acceptance among older persons may vary across seasons [15, 16].

According to the 2011 census [17], the proportion of older persons in the capital city accounted for 16.4%. Even though the highest flu immunization rates were reported among older persons in Belgrade [10], their knowledge and health beliefs relative to flu vaccination have not yet been explored. The primary aim of this study was to estimate the proportion of older persons who have ever been immunized against seasonal flu in the municipality of Vračar (Belgrade, Serbia). In addition, secondary aims were (1) to assess factors associated with flu vaccination in the population of persons over 65 years of age in the same municipality and (2) to explore the knowledge related to influenza virus and flu immunization. Finally, information obtained in this study could be used in a broader public health context in terms of designing evidence-informed interventions to increase the uptake of flu vaccine among older persons.

## Methods

### Setting

In the Republic of Serbia, health care system is financed by mandatory contributions to a social health insurance scheme. Delivery of health care is set according to three levels: primary, secondary, and tertiary. Primary level of health care is provided in 157 state-owned Community Health Centers, which may be relatively large structures, including a number of attached ambulatories, pharmacies, and institutes. One Community Health Center covers the territory of usually one or, in some cases, more municipalities or towns, with a network of outpatient facilities. Ambulatories of the Center are staffed according to the size and needs of the population they serve, varying from several full-time teams of doctors and nurses, dentists, and pharmacists working in shifts to one or two weekly doctor visits in remote ambulatories [18].

According to norms, all citizens should have access to a Community Health Center or ambulatory within 15-min travel distance. Activities of the Community Health Center are based on the chosen doctor scheme, which requires citizens to register with a primary care physician of their choice. The team of chosen physicians in the Center consists of general practitioners and occupational medicine specialist for the adult population, pediatrician for children of preschool and school age (including antenatal care, immunizations, preventive programs in the health care of children), gynecologists for women over 15 years, and dentists. In 2009, over 75% of the population has registered with a chosen doctor, who is coordinating health care across levels and being accountable for it [18, 19].

Belgrade, the capital of the Republic of Serbia, with a population of around 1.6 million inhabitants is divided in relation to geographical position, in 17 urban municipalities. The central urban municipality of Vračar was selected for this study because of the highest proportion of older persons registered as community dwellers (20.8%) of all 17 municipalities [17]. In addition, Community Health Center was chosen for recruitment of study participants because of the most convenient access to older population in the community.

### Subjects

From mid-November 2012 to mid-January 2013, persons above 65 years of age who visited the Community Health Center “Vračar” during working week (Monday to Friday) were recruited. According to the 2011 census [17], 11,384 persons above age 65 were registered as permanent community dwellers in Belgrade municipality of Vračar. Over the study period, 5287 persons above 65 years of age paid a total of 9012 visits to the Community Health Center “Vračar.” To collect a representative sample of older persons, we calculated the sample size. Taking into consideration the expected prevalence of flu immunization among older persons of 50%, size of older population in Vračar (roughly 11,000), confidence level of 95%, and confidence interval of 5, the calculated sample size was 372 persons [20]. To collect this sample, we assumed that 30% would decline participation in the survey [21]. Because of this, a total of 480 consecutive persons during the period mentioned above were approached in the premises of the Community Health Center by the investigators (TG, NK, SN, IK, TG) and were invited to participate in the survey. Older persons who visited the Center in the study period did so to obtain an extension for their drug prescriptions or to have a general practitioner’s consultation.

Inclusion criteria were the following: consent to participate in the study and a Mini-Mental State Examination (MMSE) [22] score of 24 and above. After obtaining a written consent, participants were tested for potential cognitive impairment using MMSE.

Participation in the study was anonymous. Ethical approval for the study was obtained from the Institutional Review Board of the Faculty of Medicine, University of Belgrade. Participants signed an informed consent prior to enrollment in the study.

### Instrument

Data were collected in waiting rooms of the Community Health Center (division of general practice). An anonymous questionnaire, comprising 33 questions, was used in data collection (Annex 1). Demographic and clinical characteristics referred to age, gender, marital status, place of residence, level of education, employment status, monthly income (expressed in Euros), and medical history (presence of major cardiovascular, respiratory, gastrointestinal, metabolic, urogenital, neurologic, psychiatric, visual, audio-vestibular, and orthopedic disorders). Because cardiovascular, respiratory, and metabolic diseases represent known risk factors for developing severe flu, we categorized the presence of these conditions as “chronic diseases indicative of flu immunization”.

Set of items exploring knowledge was modified from Sočan et al. [23] and Yanni et al. [24] and consisted of 14 items. Each item offered several answer options (from minimum of two to a maximum of eight given answer options). Participants were required to encircle the most appropriate answer. Three questions (regarding mode of transmission, indications, and contraindications for flu immunization) provided 2 correct answers. Each correct answer in the set of items exploring knowledge of older persons about flu and flu immunization was awarded 1 point. Although this section consisted of 14 items, 3 questions provided 2 correct answers. Hence, 3 questions were awarded 2 points. Therefore, the total knowledge score represented a range between 0 as minimum and 17 points as maximum. Consequently, the score range was divided into four levels in line with these cutoff values: 0–4 = poor, 5–8 = moderate, 9–13 = good, 14–17 = excellent. In addition, using a set of reasons, modified from Toy et al. [25], we evaluated reasons commonly given for uptake or not of the flu vaccine by older persons in Vračar municipality.

### Data analysis

To assess potential differences in relation to previous flu immunization experience, the study participants were divided into two groups: “ever vaccinated” and “never vaccinated.” Differences in examined variables were assessed by using the Mann-Whitney *U* test for two independent samples and chi-square test. Cutoff values were determined according to the median (for continuous variables) or according to the highest frequency (for categorical variables).

Moreover, we tested the interaction between the presence of chronic diseases indicative of flu immunization and demographic and clinical characteristics of study participants. To determine factors associated with flu vaccination among older persons, we performed univariate logistic regression analyses. The dependent variable in these models was being ever vaccinated against flu (yes/no). Independent variables (risk factors) were socio-demographic (gender, age, years of formal education, marital status household monthly income, place of residence, number of additional household members) and health-related (number of comorbidities, presence of chronic diseases indicative of flu immunization, amount of medications used daily). All variables univariately statistically significant and marginally significant (according to Hosmer and Lemeshow variables with *p* < 0.250) entered the multivariate logistic regression models (Enter method). Statistical analysis was performed in SPSS 17.0 statistical software package (SPSS Inc., Chicago, IL, U.S.A.).

## Results

Of 480 older persons, 126 individuals (26.3%) refused to participate. A total of 354 older persons comprised the study sample (response rate 73.7%). Considering the total number of community dwellers above 65 years of age in the municipality of Vračar (11,384 inhabitants), this sample represents 3.1% of the total older population.

Of 354 older persons, 56.2% (199) were women and 43, 8% (155) were men. The mean age was 73.7 ± 5.6 years (the youngest was 66, while the oldest was 89). One half (52.3%) have never been vaccinated. Of those who were ever vaccinated (47.7%), approximately one third (29.1%) got immunized every season. However, during ongoing flu vaccination season, more than one half of seniors (60.0%) refused to get the shot, although 85.7% were aware that they should undergo flu immunization regularly.

Basic demographic characteristics of the seniors according to the history of flu immunization are presented in Table 1. Older persons who had ever vaccinated been against seasonal flu were statistically significantly more educated (*Z* = − 3.913, *p* = 0.001), resided more often in apartments (*χ*^2^ = 3.928, *p* = 0.047), and were prescribed more medications (*Z* = − 2.609, *p* = 0.009). However, there were no differences in terms of earlier flu immunization among men and women (*χ*^2^ = 0.426, *p* = 0.513). Also, older persons who had been vaccinated were of similar age as never vaccinated (*Z* = − 1.313, *p* = 0.189) and had similar number of household members (*Z* = − 0.699, *p* = 0.484) as well as monthly income per household (*Z* = − 1.778, *p* = 0.075). Study participants did not differ according to the number of chronic diseases indicative of flu vaccination (*Z* = − 0.455, *p* = 0.649). Those who had been vaccinated demonstrated statistically significantly higher flu-related knowledge as opposed to those who have never got the shot (*Z* = − 4.999, *p* < 0.001). We did not observe the difference in knowledge scores between men and women (*Z* = − 0.004, *p* = 0.997).

**Table 1:** Basic demographic and clinical characteristics of older persons and their knowledge score according to vaccination status (*N* = 354)

Variable	Influenza immunization status		Test value	*p*
	Ever vaccinated (%)	Never vaccinated (%)		
Gender				
Male	78 (46.1)	79 (42.7)	0.426*	0.513
Female	91 (53.9)	106 (57.3)		
Age	74.2 ± 5.9	73.3 ± 5.3	− 1.313	0.189
Marital status				
Married	97 (57.1)	93 (50.5)	1.260*	0.060
Other (single, divorced, widowed)	73 (42.9)	91 (49.5)		
Place of residence				
Apartment	143 (84.6)	141 (76.2)	3.928	0.047
House	26 (15.4)	44 (23.8)		
Additional members of the household	2.2 ± 1.2	2.4 ± 1.4	− 0.699	0.484
Years of schooling	13.4 ± 2.8	12.0 ± 3.2	− 3.913	0.001
Monthly income per household (Euros)	484 ± 330	440 ± 244	− 1.778	0.075
Chronic diseases indicative of flu immunization	1.3 ± 0.7	1.2 ± 0.7	− 0.455	0.649
Prescribed medication intake per day	3.4 ± 2.1	2.8 ± 1.8	− 2.609	0.009
Knowledge score	12.8 ± 2.1	11.5 ± 2.6	− 4.999	0.001

*Chi-square (*χ*^2^ value), all others denote Mann-Whitney *U* test (*Z* value)

Table 2 displays flu-related knowledge level according to previous flu vaccination experience. When items in the knowledge section were analyzed separately, we observed that of all items, questions related to flu treatment, indications, and contraindications for vaccine administration yielded the least proportion of correct answers. Namely, almost one half of seniors (44.9%) considered using antibiotics in flu therapy, while the most appropriate answer was “use of antipyretics.” Only one third (35.0%) knew both indications for flu immunization (i.e., being older than 65 and suffering from chronic diseases). Additionally, 45.3% supposed that pollen allergy or previous flu infection represented a contraindication to vaccination. In contrast, correct answers to this question were “allergy to vaccine components” and “persons who have fever.”

**Table 2:** Distributions of older persons according to immunization status and knowledge level (numbers in brackets in knowledge score column indicate the number of correct answers)

Knowledge level	Ever vaccinated (%)	Never vaccinated (%)	Overall (%)
Poor (0–4)	1 (0.6)	4 (2.2)	5 (1.4)
Moderate (5–8)	6 (3.5)	15 (8.1)	21 (5.9)
Good (9–13)	99 (58.6)	122 (65.9)	221 (62.4)
Excellent (14–17)	63 (37.3)	44 (23.8)	107 (30.2)

To verify whether or not the presence of chronic diseases indicative of flu immunization had an influence on demographic and clinical variables that could be potentially associated with flu vaccination, we performed testing for interaction (see Additional file 1). We found no sufficient evidence to acknowledge the influence of chronic diseases indicative of flu immunization on selected variables (potential predictors of flu vaccination). Univariate logistic regression analysis (Table 3) showed that longer formal education, higher income level, residing in an apartment, taking more prescribed medications daily, and having higher knowledge score related to flu and flu vaccination were statistically significantly associated with flu immunization. Multivariate logistic regression analysis (Table 3) demonstrated that higher education level, taking more prescribed medications, and having higher knowledge score were independently associated with seasonal flu vaccination among older persons.

**Table 3:** Factors associated with flu vaccination among older persons

Risk factor	Count	Univariate logistic regression			Multivariate logistic regression		
		OR	95% CI	*p*	OR	95% CI	*p*
Gender							
Males	155	1.15	0.75–1.76	0.513			
Females	199						
Age (years)							
> 74	166	1.23	0.81–1.88	0.326			
66–73	187						
Years of schooling							
> 12	143	2.02	1.31–3.13	0.001	1.87	1.13–3.08	0.014
≤ 12	205						
Household monthly income (Euro)							
> 388	154	1.57	1.01–2.46	0.045	1.26	0.73–2.16	0.410
≤ 388	162						
Marital status							
Married	190	1.34	0.88–2.04	0.177	1.27	0.74–2.17	0.378
Other	164						
Place of residence							
Apartment	286	1.74	1.01–3.01	0.046	1.98	0.93–3.86	0.134
House	68						
Additional members of the household							
0–2	286	1.20	0.76–1.88	0.430			
≥ 3	68						
Presence of chronic disease indicative of flu immunization							
yes	306	0.95	0.51–1.75	0.857			
no	48						
Amount of prescribed medications used daily							
≥ 4	135	1.55	1.01–2.40	0.047	1.71	1.06–2.77	0.029
0–3	219						
Flu knowledge score							
≥ 13	106	1.87	1.17–2.96	0.008	1.80	1.08–2.98	0.023
< 13	248						

*OR* odds ratio, *CI* confidence interval

**Fig. 1:**
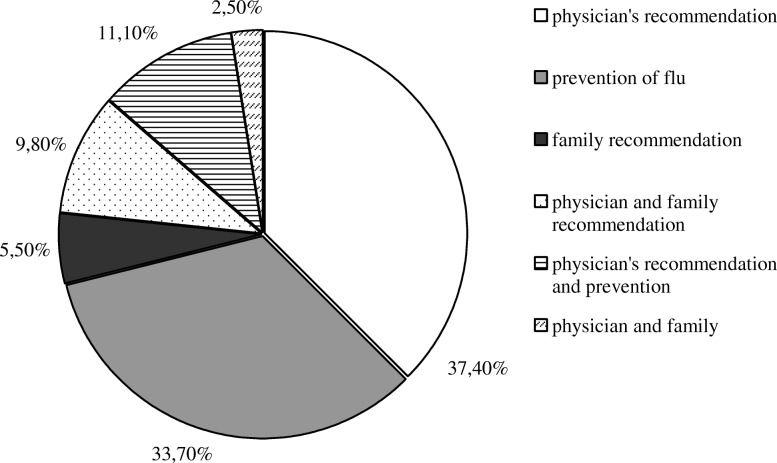
Distribution of reasons for flu vaccination acceptance among older persons

**Fig. 2:**
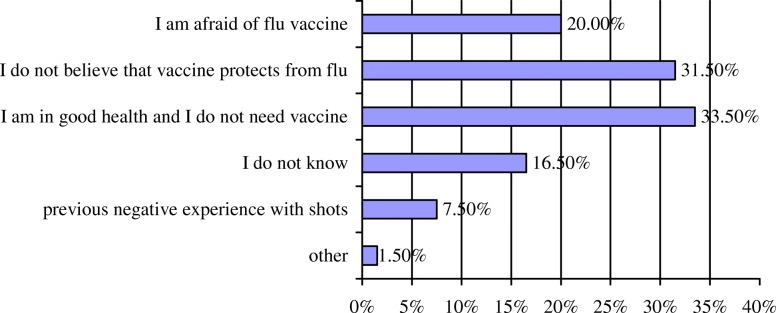
Distribution of reasons for non-compliance with flu vaccination among older persons based on their opinions

Of those who have ever been vaccinated, almost one half (49.7%) accepted flu immunization because of doctor’s recommendation, while another 47.3% considered that seasonal flu vaccine prevents the flu. Also, 17.8% of older persons confirmed that they had been immunized because of family recommendation (Fig. 1). Reasons for vaccine refusal are given in Fig. 2. The highest proportion of reasons not to take the flu vaccine accounted for the assertions “I am in good health and I do not need a vaccine” (33.5%) and “I do not believe vaccine protects from flu” (31.5%). Small proportion of participants (7.5%) revealed that they have had a negative experience with previous flu vaccinations in terms of adverse postvaccinal reactions.

## Discussion

Our study showed that the proportion of older persons ever vaccinated against seasonal flu in Vračar municipality (Belgrade) was 47.7%. This proportion is considerably lower than 75% targeted by the World Health Organization [26]. Moreover, only one third of ever vaccinated older persons is being immunized every season. The proportion of ever vaccinated older persons in our study is similar to that reported in Taiwan (43.7%, for the last analyzed vaccination season of 2008–2009) [27]. Also, slightly higher proportion (58.6%) of vaccinated seniors was documented in Spain [28]. According to reports of Mereckiene et al. [29], the highest flu vaccination coverage in European Union was observed in the Netherlands (82.1%) and in the UK (75%), reaching proposed coverage threshold of 75% by 2010 [30]. However, in some countries of the Mediterranean and Eastern Europe, such as Turkey [31], Poland [32], and Romania [29], extremely low prevalence has been reported (5.9%, 13.4%, and 18.3%, respectively). At a global level, the highest flu immunization coverage in a population of older persons was observed in North America with 68.9% [33] and Brazil with 73% [34], approaching recommended WHO rate [30].

Low acceptance of seasonal flu vaccination could be explained by several factors. Firstly, a substantial proportion of older persons in our research did not believe in the effectiveness of the flu vaccine, expressing doubt in its quality and origin of vaccine components. Personal beliefs have also been reported as the most common reason for non-compliance in Turkey. Namely, Ciblak [31] highlighted that approximately one third of Turkish older adults believe that the flu vaccine is not effective and, therefore, refuse immunization. Secondly, we observed that some older persons were afraid of seasonal flu vaccination, expressing concern that flu vaccination might, in fact, cause flu. This issue, i.e., non-compliance with flu vaccination as a result of fear of side-effects, has been documented in industrialized countries, such as the Netherlands [35] and Canada [36], as well. Thirdly, older persons in Vračar municipality were compelled not to get vaccinated because they considered themselves to be in “good health.” Similarly, a qualitative study among older Canadians implied that belief in resilience represents a major barrier to vaccination [36]. Yu et al. [37] emphasized that perception of own susceptibility in older population is significantly predictive of flu immunization. Our findings suggested that health services should develop strategies to reshape health beliefs with the aim of securing positive attitudes towards seasonal flu immunization vaccination.

Although the highest proportion of participants, regardless of flu vaccination status, demonstrated good level of flu-related knowledge, certain gaps and misconceptions were noted. For example, high proportion of wrong answers in our study was related to the use of antibiotics in flu treatment. Even though in the Republic of Serbia antibiotics cannot be bought without prescription, it is likely that misconceptions concerning when and for what reason antibiotics are used (i.e., in every type of infection) have led study participants to consider that this type of medication is appropriate in flu treatment. On the other hand, only 0.3% of older persons in Vračar indeed practice self-medication with antibiotics [38]. Another mistaken belief among older persons was related to the notion that having pollen allergy could be a reason to withhold seasonal flu immunization. We presume that some older persons have clinically manifested seasonal allergies and therefore believe that in such circumstances administration of flu vaccine should be restricted. In addition, the majority of study participants did not know conditions in which flu vaccine should be administered. Given these findings, it could be argued that false beliefs and lack of knowledge in some aspects likely cause low flu vaccination acceptance. Because of this, promotion of seasonal flu vaccination through counseling in Community Health Centers and through media could be helpful in improving flu vaccination uptake among the older population.

Independent predictors of flu immunization among older residents of Vračar municipality were having more years of formal education, having more knowledge related to flu vaccination, and taking more medications. Education level has been consistently reported in the literature as one of the strongest predictors of flu vaccine acceptance [33, 39, 40]. It is likely that older persons who have higher level of education are indeed more willing to accept healthy behaviors. Also, it is possible that these individuals are more aware of potential risks associated with influenza virus infection and therefore respond to immunization. This could be, again, related to the fact that over the period of schooling they have adopted skills as to how to seek after (health) information. As expected, people with higher levels of education are likely to know more about flu and flu immunization. In addition, higher knowledge on the subject may facilitate acceptance of vaccination. Even though higher flu-related knowledge could result in higher likelihood of flu vaccine acceptance, it is not clear whether this association could be interpreted in the opposite direction. Specifically, it is possible that persons who accept flu immunization inform themselves more than those who do not and subsequently demonstrate higher level of knowledge relative to flu. For example, investigation among health-care workers in the UK demonstrated that the lowest knowledge scores were attributed to those who had never been vaccinated as opposed to higher scores of those previously vaccinated [41]. Lastly, taking more medications could be interpreted as proxy for having more chronic conditions. Having multiple chronic conditions has been strongly associated with higher likelihood of flu vaccination [33, 42] due to flu-related complications [4–6]. However, because no association was established between having known risk factors (chronic conditions indicative of flu vaccination) and being immunized against flu, we assume that the acceptance of flu vaccine in older population of Vračar municipality is more likely attributed to personal beliefs, perceptions of risk, higher education level, and higher knowledge on the subject regardless of underlying medical conditions. Finally, we cannot exclude that this might be a chance finding because of study limitations.

Several study limitations need to be addressed. Firstly, our study could have benefitted from a larger sample size. Sampling for the study was limited to one Community Health Center located in the central urban municipality. It is possible that inclusion of older persons who visit suburban Centers would have decreased the proportion of vaccinated individuals. Specifically, older persons residing in central municipalities have more opportunities to interact with peers through social clubs or with neighbors because in this area population density is higher and distance from various city services is considerably shorter compared with suburban areas. Therefore, it is possible that the proportion of vaccinated older persons in suburban municipalities is lower. Sampling strategy might have introduced selection bias, given that persons who regularly visit Community Health Center are either suffering from chronic conditions or take more precautions regarding health compared to older persons who do not practice equal health behavior. Secondly, we should consider potential information bias, because flu vaccination status was self-reported. Although we aimed at excluding persons with minor/major cognitive impairment using the MMSE, recall bias cannot be ruled out. Hence, it is probable that the actual proportion of vaccinated older persons against seasonal flu is lower. Also, the survey was conducted during vaccination season when more information related to seasonal flu is generally available, which may suggest that the study participants demonstrated higher knowledge level on the subject. Thirdly, this study was limited by the use of cross-sectional design. Hence, to elucidate whether or not older persons who demonstrated higher knowledge level increase their knowledge after immunization or due to higher knowledge they comply with immunization, a prospective cohort study design with follow-up across several vaccination seasons would be more appropriate.

## Conclusions

In summary, this was the first study of flu vaccination knowledge and practices in Serbia. Our findings indicate that the proportion of older persons vaccinated against flu was lower than that in industrialized countries. Factors associated with flu immunization among older persons indicate that health care sector requires well-coordinated promotion campaigns to enhance acceptance of flu vaccination and therefore secure higher vaccination coverage in this population group. Furthermore, organized immunization counseling, particularly during flu vaccination season, in the Community Health Centers as well as in social clubs for older adults, would provide accurate, evidence-based information in order to transform misbeliefs, prejudice, and negative attitude towards vaccination. To achieve long-term results, mobilization of media (in form of video clips, billboards, banners, leaflets, etc.) and government institutions would be beneficial for additional dissemination of adequate data with the goal to improve flu-related knowledge in older population groups.

## Additional file

Additional file 1: Exploration of interaction by means of logistic regression model between chronic diseases indicative of flu immunization and selected variables. (DOC 41 kb)
