# Supplementary material for: Flu vaccination among older persons: study of knowledge and practices
Source: J Health Popul Nutr. 2019 Jan 3;38:2. doi: 10.1186/s41043-018-0159-8 (PMC6318864; doi:10.1186/s41043-018-0159-8)
Supplement: Supplementary file 1 — Exploration of interaction by means of logistic regression model between chronic diseases indicative of flu immunization and selected variables. (DOC 41 kb) [file 41043_2018_159_MOESM1_ESM.doc]

Additional file 1 Exploration of interaction by means of logistic regression model between chronic diseases indicative of flu immunization and selected variables

| Variable 1 (V1) | Multiplication  procedure | Variable 2 (V2) | Product (V1xV2)  p-value  in the model |
| --- | --- | --- | --- |
| Gender | X | CDIFI | 0.902 |
| Age | X | CDIFI | 0.158 |
| Years of schooling | X | CDIFI | 0.842 |
| Income | X | CDIFI | 0.137 |
| Marital status | X | CDIFI | 0.782 |
| Place of residence | X | CDIFI | 0.483 |
| Members of household | X | CDIFI | 0.818 |
| No. of medications | X | CDIFI | 0.743 |
| Flu knowledge score | X | CDIFI | 0.088 |

CDIFI – chronic diseases indicative of flu immunization (cardiovascular, respiratory and metabolic conditions)

Logistic regression model

outcome/dependent variable: being ever vaccinated against seasonal flu

determinants/independent variables: Variable 1 + Variable 2 + Variable 1 x Variable 2
